# Correlates of prolonged length of stay after endoscopic transsphenoidal surgery for pituitary adenomas: varying definitions and non-clinical factors

**DOI:** 10.1007/s11102-024-01483-z

**Published:** 2025-01-25

**Authors:** Khushi H. Shah, Nikola Susic, Nicholas V. DiStefano, Maxon V. Knott, Adham M. Khalafallah, Victor M. Lu, Carolina G. Benjamin, Ashish H. Shah, Zoukaa B. Sargi, Ricardo J. Komotar, Michael E. Ivan

**Affiliations:** 1 Department of Neurological Surgery, University of Miami Miller School of Medicine, 1095 NW 14th Terrace, 2nd Floor, Miami, Fl 33136 USA; 2 Department of Otolaryngology, University of Miami, Miller School of Medicine, Miami, FL USA; 3 Sylvester Comprehensive Cancer Center, University of Miami Health System, Miami, FL USA

**Keywords:** Hospital length of stay, Prolonged length of stay, Pituitary adenoma, Endoscopic transsphenoidal surgery

## Abstract

**Purpose:**

Prolonged length of stay (PLOS) can lead to resource misallocation and higher complication risks. However, there is no consensus on defining PLOS for endoscopic transsphenoidal pituitary surgery (ETPS). Therefore, we investigated the impact of varying PLOS definitions on factors associated with PLOS in patients undergoing ETPS.

**Methods:**

We conducted a retrospective review of patients with pituitary adenomas who underwent ETPS at our institution from 2012 to 2023. Patients were divided into non-PLOS and PLOS groups based on varying definitions of PLOS: > median, > 4 days, > 75th percentile, and > 90th percentile. Bivariate statistical analyses were conducted using Fisher’s exact test, chi-square test, and t-tests. Univariate and multivariate logistic regression identified significant predictors for each PLOS definition.

**Results:**

Our cohort (*n* = 808) had a mean age of 54.37 ± 16.06 years, 50.43% male, and a median LOS of 3 days. The 75th and 90th percentiles of LOS were 4 and 6 days, respectively. The way PLOS was defined influenced associated factors identified. Preoperative KPS score, non-private insurance, and non-home discharge disposition were associated with PLOS across all definitions used (*p* < 0.05). Increased preoperative tumor volumes and postoperative hyponatremia were associated with PLOS only when defined by the 75th and 90th percentiles (*p* < 0.05). Non-White race and low income were significantly associated with PLOS > median while intraoperative CSF leak was a significant predictor for PLOS > 90th percentile (*p* < 0.05).

**Conclusion:**

Our study highlights the variability in predictors of PLOS based on its definition and emphasizes the role of non-clinical factors on LOS.

## Introduction

Hospital length of stay (LOS) is a critical health metric often used as an indicator of efficient hospital management [1]. Various clinical databases, including the University Health System Consortium, National Trauma Data Bank, and National Surgical Quality Improvement Program (NSQIP), consider LOS a key measure of the quality of patient care provided [2]. The assumption is that a shorter LOS reflects more efficient and effective patient care [1, 2]. Additionally, reducing LOS improves bed turnover, enabling hospitals to better accommodate the growing demand for elective and emergency admissions [1]. Conversely, prolonged LOS (PLOS) can lead to a misallocation of hospital resources and an increased risk of patient complications, such as hospital-acquired infections and falls [3–5]. Despite its importance, there is no consensus in the literature on what constitutes PLOS. Large studies in non-neurosurgical literature have variously defined PLOS as either exceeding the mean LOS or being greater than the 75th percentile [2, 6, 7].

Transsphenoidal surgery is the first-line treatment for most pituitary adenomas (PAs), with a growing trend toward endoscopic transsphenoidal pituitary surgery (ETPS) over the traditional microscopic approach [8–11]. The median LOS after ETPS is typically around 3 days [12, 13]. However, studies evaluating PLOS after ETPS have used varying definitions, such as greater than the median, more than 4 days, exceeding the 75th percentile, or being greater than the 90th percentile [12–16]. Three large studies based on the NSQIP and National Inpatient Sample (NIS) databases examined factors related to PLOS after ETPS but lacked granular information on preoperative characteristics, tumor features, and postoperative outcomes [12, 15, 17]. Only two single-institution studies with smaller number of patients from the United States have evaluated predictors of PLOS [14, 16], both using different definitions of the term and failing to consider non-clinical factors such as insurance status and discharge disposition which can significantly impact LOS [2]. Additionally, PLOS is associated with increased costs for both patients and hospitals [18]. For example, a reduction in mean hospital stay from 4.46 to 3.32 day after implementing a streamlined care pathway for patients undergoing ETPS led to a cost savings of $6,036 per patient [19].

Given this context and the limitations in the current literature, we conducted the present study to evaluate various patient demographic, perioperative, and non-clinical factors that are associated with PLOS. We also explored different definitions of PLOS to examine how they influence its predictive factors. Our primary hypothesis is that the factors influencing PLOS vary depending on the definitions used and the secondary hypothesis is that non-clinical factors play a significant role in determining LOS after ETPS.

## Methods

### Patient

After Institutional Review Board Approval (IRB no. 20160437), a retrospective chart review was conducted of patients who underwent ETPS at our tertiary care institution from 2012 to 2023. All patients were evaluated preoperatively with magnetic resonance imaging (MRI) and hormonal workup. For the surgery, a two-surgeon approach was employed, involving neurosurgery and otolaryngology with active involvement of residents and fellows. Surgical technique employed [20] and postoperative patient management [21] have been described by us previously. Patients with age ≥ 18 years, a histopathological diagnosis of PA, and data on LOS were included in this study. Patients with other pituitary tumors were excluded. Informed consent was waived due to the retrospective nature of this study.

### Data collection

Patient demographics were collected, including age at surgery, gender, race, ethnicity, insurance status, and median household income as estimated by cross-referencing patient’s zip code at the time of surgery with the US Census Bureau data. Low income was defined as lowest quartile of estimated household income. Preoperative clinical data, including modified 5-item frailty index (mFI-5) factors and score, additional comorbidities such as atrial fibrillation, long-term anticoagulant use, and obstructive sleep apnea, karnofsky performance score (KPS), apoplexy, history of radiation to the pituitary area, were collected. MRI scans were reviewed for preoperative tumor volume, chiasm compression, and cavernous sinus invasion as defined as Knosp grade 3 and 4 [22]. Tumor volumes were calculated using the ellipsoid formula (length x width x height/2), based on the greatest dimensions in axial, coronal, and sagittal planes from T1 weighted contrasted MRI images. Intraoperative data included surgery duration, intraoperative CSF leak, and use of lumbar drain. 24-hour postoperative MRI scans were reviewed to determine residual tumor volume and extent of resection (EOR). Data on outcomes included LOS in days and hours, postoperative complications before discharge including CSF leak, diabetes insipidus (DI), hyponatremia, deep vein thrombosis, pulmonary embolism, and urinary tract infection. Discharge disposition was obtained for each patient and home and home health were classified under “home” discharge while other facilities were classified under “non-home”.

### Statistical analysis

Patients were divided into standard LOS (non-PLOS) and PLOS groups based on varying definitions of PLOS. PLOS was assessed at multiple definitions including greater than median LOS, greater than 4 days, greater than 75th percentile of LOS, and greater than 90th percentile of LOS. We also calculated median LOS in patients with complex tumors such as giant pituitary adenoma > 4 cm maximum diameter or those with cavernous sinus invasion.

Descriptive statistics were performed to compare variables across non-PLOS and PLOS cohorts using each definition. Comparison of categorical variables between the study and control cohorts was performed using chi-square and Fisher exact tests, as appropriate. Continuous variables were compared using either Student’s t-test or Welch’s t-test depending on the equality of variance tested via Levene’s test. Mean and standard deviation were reported for all continuous variables, except for KPS and mFI-5, where median and interquartile range (25-75th percentile) were used due to non-normal distribution.

Univariate and multivariate logistic regression analyses were conducted to identify predictors of PLOS for each definition. Significant variables (*p* < 0.05) upon univariate analysis with adequate sample sizes and those deemed clinically relevant were included in the multivariate model. Final variable selection included testing for assumptions, correlation analysis, multicollinearity, stepwise selection, and goodness of fit. The final variables included in the multivariate model for all definitions were non-white race, non-private insurance, preoperative KPS score, chiasm compression, cavernous sinus invasion, preoperative tumor volume, intraoperative CSF leak, EOR, postoperative hyponatremia, and non-home discharge disposition. Statistical significance was set at a *p* value < 0.05 for all analyses. Statistical analyses were performed using Python version 3.11.5 for MacOS.

## Results

During the study period, out of 1070 patients who underwent ETPS at our institution, 808 met the inclusion criteria. This cohort had an average age of 54.37 ± 16.06 years, 50.43% male, and a median length of stay of 3 [IQR: 3–4] days. The 75th percentile and 90th percentile of LOS were 4 and 6 days, respectively. In subgroup of patients with giant pituitary adenoma > 4 cm in maximum diameter (*n* = 56), we noted that the median LOS to be 4 [IQR 3–6] days. Additionally, the median LOS in patients with tumors with cavernous sinus invasion was 3 [IQR 3–4] days. All patients were divided into non-PLOS and PLOS cohorts depending on four different definitions of PLOS: > median, > 4 days, > 75th percentile and > 90th percentile of LOS. In our study, the > 75th percentile and > 4 day definitions of PLOS were the same.

### PLOS as greater than median LOS

In defining PLOS as > median LOS (3 days), 256 patients met the criteria. Preoperative and adenoma characteristics for each PLOS definition are detailed in Table 1. There was no significant difference in age, gender, ethnicity, history of radiation, and apoplexy between groups. The PLOS group had greater rates of non-White race (41.3% vs. 29.3%), non-private insurance (50.6% vs. 36.9%), and individuals with lowest quartile of estimated income (30.9% vs. 22.6%) compared to the non-PLOS group (*p* < 0.001). Although the median preoperative KPS was similar in both groups, the difference was found to be statistically significant. Patients with PLOS had higher rates of preoperative visual disturbances, headaches, and comorbidities such as hypertension and anticoagulant use (*p* < 0.05). Regarding adenoma characteristics, the PLOS group had increased cavernous sinus invasion (48.0% vs. 38.0%, *p* = 0.009) and preoperative tumor volume (8.98 ± 15.37 cm³ vs. 4.93 ± 6.51 cm³, *p* < 0.001)(Table 2). In terms of operative characteristics, patients with PLOS experienced longer surgeries, increased rate of lumbar drain use, and higher residual tumor volume (*p* < 0.05). The PLOS group also had significantly lower EORs compared to its counterpart. While there was no difference in intraoperative CSF leak between groups (*p* = 0.118), patients with PLOS experienced higher rates of postoperative CSF leak (*p* < 0.001) and hyponatremia (*p* < 0.001). Finally, non-home discharge disposition was more common in patients experiencing PLOS (*p* < 0.001).

**Table 1:** Patient demographics and adenoma characteristics across varying definitions of PLOS

PLOS definition	LOS > median			LOS > 75th percentile			LOS > 90th percentile		
Variables	Non-PLOS (*n* = 552)	PLOS (*n* = 256)	*P* value	Non-PLOS (*n* = 672)	PLOS (*n* = 136)	*P* value	Non-PLOS (*n* = 746)	PLOS (*n* = 62)	*P* value
**Patient demographics**									
Age (years), mean, SD	54.83 ± 16.23	53.45 ± 15.77	0.257	54.42 ± 16.17	54.29 ± 15.73	0.931	54.35 ± 16.14	54.90 ± 15.61	0.796
Gender, male	281 (50.9%)	126 (49.2%)	0.711	332 (49.4%)	75 (55.1%)	0.260	372 (49.9%)	35 (56.5%)	0.387
Ethnicity, hispanic	208 (37.7%)	97 (37.9%)	0.643	251 (37.4%)	54 (39.7%)	0.590	282 (37.8%)	23 (37.1%)	0.230
Race:			**0.001**			0.155			0.425
White	383 (70.7%)	142 (58.7%)		448 (68.1%)	77 (61.1%)		492 (67.4%)	33 (61.1%)	
Non-white	159 (29.3%)	100 (41.3%)		210 (31.9%)	49 (38.9%)		238 (32.6%)	21 (38.9%)	
Insurance:			**< 0.001**			**< 0.001**			**< 0.001**
Private	345 (63.1%)	123 (49.4%)		414 (62.5%)	54 (40.3%)		452 (61.4%)	16 (26.7%)	
Non-private	202 (36.9%)	126 (50.6%)		248 (37.5%)	80 (59.7%)		284 (38.6%)	44 (73.3%)	
Estimated income, mean, SD	76456.45 ± 24539.34	71657.93 ± 22087.52	**0.009**	75799.59 ± 24231.75	70667.01 ± 21650.03	**0.026**	75703.61 ± 23988.50	65829.29 ± 20719.61	**0.002**
Low income	120 (22.6%)	75 (30.9%)	**0.017**	152 (23.5%)	43 (33.6%)	**0.022**	169 (23.6%)	26 (44.1%)	**0.001**
Preop KPS, mean, SD	90 (80–90)	90 (80–90)	**< 0.001**	90 (80–90)	90 (80–90)	**< 0.001**	90 (80–90)	80 (80–90)	**< 0.001**
BMI, mean, SD	29.81 ± 6.20	30.31 ± 7.43	0.321	29.92 ± 6.28	30.18 ± 8.10	0.681	29.89 ± 6.26	30.89 ± 9.95	0.254
Comorbidities									
DM	256 (46.4%)	112 (43.8%)	0.534	310 (46.1%)	58 (42.6%)	0.516	342 (45.8%)	26 (41.9%)	0.645
HTN	100 (18.1%)	69 (27.0%)	**0.005**	130 (19.3%)	39 (28.7%)	**0.020**	154 (20.6%)	15 (24.2%)	0.619
Atrial fibrillation	19 (3.5%)	14 (5.5%)	0.245	22 (3.3%)	11 (8.1%)	**0.019**	26 (3.5%)	7 (11.3%)	**0.008**
Obstructive sleep apnea	47 (8.5%)	12 (4.7%)	0.155	52 (7.7%)	7 (5.1%)	0.380	54 (7.2%)	5 (8.1%)	1.000
Long term coagulation	13 (2.4%)	8 (3.1%)	**0.047**	15 (2.2%)	6 (4.4%)	0.245	17 (2.3%)	4 (6.5%)	0.117
mFI-5	20 (0–20)	20 (0–40)	0.061	20 (0–20)	20 (0–40)	**0.029**	20 (0–20)	20 (0–20)	0.434
Current smoking	40 (7.3%)	23 (9.2%)	0.431	51 (7.6%)	12 (9.0%)	0.710	61 (8.2%)	2 (3.3%)	0.270
Hx of radiation	7 (1.3%)	3 (1.2%)	1.000	8 (1.2%)	2 (1.5%)	1.000	10 (1.3%)	0 (0.0%)	0.749
Preop visual disturbances	245 (44.4%)	158 (61.7%)	**< 0.001**	303 (45.1%)	100 (73.5%)	**< 0.001**	352 (47.2%)	51 (82.3%)	**< 0.001**
Preop headache	129 (23.4%)	84 (32.8%)	**0.006**	165 (24.6%)	48 (35.3%)	**0.013**	186 (24.9%)	27 (43.5%)	**0.002**
Apoplexy	31 (5.6%)	13 (5.1%)	0.883	36 (5.4%)	8 (5.9%)	0.969	38 (5.1%)	6 (9.7%)	0.216
**Adenoma characteristics**	112 (20.3%)	64 (25.0%)	0.156	148 (22.0%)	28 (20.6%)	0.798	167 (22.4%)	9 (14.5%)	0.200
Functional adenoma	33 (6.0%)	32 (12.5%)	**0.002**	48 (7.1%)	17 (12.5%)	**0.055**	59 (7.9%)	6 (9.7%)	0.803
ACTH	45 (8.2%)	14 (5.5%)	0.223	53 (7.9%)	6 (4.4%)	0.215	57 (7.6%)	2 (3.2%)	0.303
GH	50 (9.1%)	20 (7.8%)	0.652	63 (9.4%)	7 (5.1%)	0.152	69 (9.2%)	1 (1.6%)	0.069
PRL	350 (64.0%)	181 (71.3%)	0.052	425 (63.8%)	106 (78.5%)	**0.001**	476 (64.4%)	55 (88.7%)	**< 0.001**
Chiasm compression	497 (90.0%)	231 (90.2%)	1.000	607 (90.3%)	121 (89.0%)	0.745	670 (89.8%)	58 (93.5%)	0.468
Macroadenoma	210 (38.0%)	123 (48.0%)	**0.009**	264 (39.3%)	69 (50.7%)	**0.017**	298 (39.9%)	35 (56.5%)	**0.016**
Cavernous sinus invasion	4.93 ± 6.51	8.98 ± 15.37	**< 0.001**	5.27 ± 8.13	10.90 ± 16.90	**< 0.001**	5.57 ± 8.54	13.96 ± 21.42	**< 0.001**
Preop tumor volume (cm^3^), mean, SD	112 (20.3%)	64 (25.0%)	0.156	148 (22.0%)	28 (20.6%)	0.798	167 (22.4%)	9 (14.5%)	0.200

Bold entries signify statistical significance, *p* < 0.05

Preop, preoperative; KPS, karnofsky performance score; BMI, body mass index; DM, diabetes mellitus; HTN, hypertension; mFI-5, modified frailty index-5; hx, history

**Table 2:** Operative characteristics and treatment outcomes across varying definitions of PLOS

PLOS definition	LOS > median				LOS > 75th percentile			LOS > 90th percentile		
Variables	Non-PLOS (*n* = 552)	PLOS (*n* = 256)	*P* value	Non-PLOS (*n* = 672)		PLOS (*n* = 136)	*P* value	Non-PLOS (*n* = 746)	PLOS (*n* = 62)	*P* value
**Operative characteristics**										
Surgery length (mins), mean, SD	254.91 ± 45.83	276.79 ± 76.52	**< 0.001**	256.18 ± 47.02		288.09 ± 91.19	**< 0.001**	258.24 ± 52.59	292.32 ± 86.94	**< 0.001**
Intraop CSF leak	295 (53.4%)	152 (59.6%)	0.118	362 (53.9%)		85 (62.5%)	0.083	405 (54.4%)	42 (67.7%)	0.057
Use of lumbar drain	2 (0.4%)	21 (8.2%)	**< 0.001**	6 (0.9%)		17 (12.5%)	**< 0.001**	16 (2.1%)	7 (11.3%)	**< 0.001**
Residual tumor volume (cm^3^), mean, SD	0.31 ± 1.33	0.88 ± 3.56	**0.001**	0.40 ± 2.25		0.93 ± 2.44	**0.014**	0.42 ± 2.24	1.27 ± 2.81	**0.005**
EOR (%), mean, SD	96.70 ± 9.41	94.58 ± 14.70	**0.014**	96.54 ± 10.61		93.46 ± 14.44	**0.004**	96.39 ± 10.93	91.72 ± 15.40	**0.002**
**Treatment outcomes**										
Length of stay (days), mean, SD	2.99 ± 0.13	6.05 ± 5.23	**< 0.001**	3.17 ± 0.40		7.86 ± 6.68	**< 0.001**	3.38 ± 0.77	10.89 ± 9.03	**< 0.001**
Length of stay (hours), mean, SD	72.28 ± 4.16	145.64 ± 125.29	**< 0.001**	76.69 ± 10.29		188.57 ± 160.25	**< 0.001**	81.76 ± 18.52	261.15 ± 216.38	**< 0.001**
Postop complications:										
Postop CSF leak	1 (0.2%)	10 (3.9%)	**< 0.001**	2 (0.3%)		9 (6.6%)	**< 0.001**	8 (1.1%)	3 (4.8%)	0.059
DI	193 (35.0%)	95 (37.1%)	0.608	233 (34.7%)		55 (40.4%)	0.237	263 (35.3%)	25 (40.3%)	0.508
Hyponatremia	114 (20.7%)	83 (32.4%)	**< 0.001**	144 (21.4%)		53 (39.0%)	**< 0.001**	167 (22.4%)	30 (48.4%)	**< 0.001**
DVT	0 (0.0%)	2 (0.8%)	0.187	0 (0.0%)		2 (1.5%)	**0.028**	1 (0.1%)	1 (1.6%)	0.357
PE	3 (0.6%)	3 (1.2%)	0.575	4 (0.6%)		2 (1.5%)	0.585	4 (0.6%)	2 (3.4%)	0.098
UTI	6 (1.1%)	3 (1.2%)	1.000	8 (1.2%)		1 (0.8%)	0.997	9 (1.2%)	0 (0.0%)	0.832
Discharge disposition:			**< 0.001**				**< 0.001**			**< 0.001**
Home	540 (100.0%)	234 (95.9%)		653 (100.0%)		121 (92.4%)		725 (99.9%)	49 (84.5%)	
Non home	0 (0.0%)	10 (4.1%)		0 (0.0%)		10 (7.6%)		1 (0.1%)	9 (15.5%)	

Bold entries signify statistical significance, *p* < 0.05

Intraop, intraoperative; EOR, extent of resection; postop, postoperative; DI, diabetes insipidus; DVT, deep vein thrombosis; PE, pulmonary embolism; UTI, urinary tract infection

### PLOS as greater than 75th percentile LOS

When defining PLOS as > 75th percentile of LOS (4 days), 136 patients experienced PLOS. There was no significant difference in age, gender, ethnicity, history of radiation and apoplexy between groups (Table 1). However, patients experiencing PLOS had higher rates of non-private insurance (59.7% vs. 37.5%, *p* < 0.001) and low income (33.6% vs. 23.5%, *p* = 0.022). Similar to the PLOS > median definition, preoperative KPS score, while similar, was found to be significantly different between groups. The PLOS group also had higher rates of comorbid conditions such as hypertension and atrial fibrillation, increased frailty as assessed by mFI-5, and higher rates of preoperative visual disturbances and headache (*p* < 0.05). They also had higher rates of chiasm compression, cavernous sinus invasion, increased preoperative tumor volumes, and reduced EOR (*p* < 0.05). Postoperative complications, such as hyponatremia (39.0% vs. 21.4%, *p* < 0.001), postoperative CSF leaks (6.6% vs. 0.3%, *p* < 0.001), and deep vein thrombosis (1.5% vs. 0.0%, *p* = 0.028) were significantly more common in the PLOS group (Table 2). Additionally, 7.6% of PLOS patients were discharged to non-home settings, compared to none in the non-PLOS group (*p* < 0.001).

### PLOS as greater than 90th percentile LOS

On expanding PLOS definition to the 90th percentile of the length of stay (> 6 days), 62 patients experienced PLOS. They had significantly increased rates of non-private insurance (73.3% vs. 38.6%, *p* < 0.001) and low income (44.1% vs. 23.6%, *p* = 0001). More patients in the PLOS had a diagnosis of atrial fibrillation as a comorbidity (11.3% vs. 3.5%, *p* = 0.008). They also reported preoperative headache (43.5% vs. 24.9%, *p* = 0.002) and visual disturbances (82.3% vs. 47.2%, *p* < 0.001). On imaging, the PLOS group experienced significantly higher rates of chiasm compression, cavernous sinus invasion, and higher preoperative tumor volumes (Table 1). Patients with PLOS had longer surgeries, increased rates of lumbar drain use, greater residual tumor volumes, and lower EOR (*p* < 0.05)(Table 2). Postoperatively, patients with PLOS experienced more hyponatremia and discharge to a non-home setting (*p* < 0.001).

### Univariate analysis

Upon univariate logistic regression analysis, preoperative factors such as non-private insurance, low income, lower preoperative KPS score, presence of preoperative visual disturbances, chiasm compression, cavernous sinus invasion, and increased preoperative tumor volume were significantly associated with PLOS across all definitions used (*p* < 0.05)(Table 3). Similarly, operative and postoperative factors such as surgery length, postoperative hyponatremia, and non-home discharge were significantly associated with PLOS across all definitions (*p* < 0.05).

**Table 3:** Univariate logistic regression analysis across varying definitions of PLOS

PLOS definition	LOS > median		LOS > 75th percentile			LOS > 90th percentile		
Variables	Odds Ratio	*P* value	Odds Ratio	*P* value	Odds Ratio		*P* value	
**Patient demographics**								
Age (years)	1.01 (1.00, 1.02)	0.057	1.00 (0.99, 1.01)	0.846	1.00 (0.99, 1.02)		0.824	
Gender, male	1.01 (0.87, 1.17)	0.878	1.05 (0.89, 1.23)	0.585	1.17 (0.92, 1.49)		0.201	
Ethnicity, hispanic	0.75 (0.56, 1.00)	**0.048**	1.01 (0.73, 1.41)	0.938	0.79 (0.48, 1.30)		0.359	
Race:								
White	Ref		Ref		Ref			
Non-white	1.79 (1.29, 2.48)	**< 0.001**	1.36 (0.97, 1.92)	0.073	1.19 (0.72, 1.97)		0.495	
Insurance:								
Private	Ref		Ref		Ref			
Non-private	1.62 (1.20, 2.20)	**0.002**	2.32 (1.66, 3.22)	**< 0.001**	3.83 (2.27, 6.46)		**< 0.001**	
Low income	1.64 (1.15, 2.35)	**0.007**	1.53 (1.06, 2.22)	**0.022**	2.36 (1.42, 3.92)		**< 0.001**	
Preop KPS	0.97 (0.95, 0.99)	**< 0.001**	0.94 (0.92, 0.96)	**< 0.001**	0.92 (0.90, 0.95)		**< 0.001**	
BMI	1.00 (0.98, 1.03)	0.815	1.00 (0.98, 1.03)	0.852	1.01 (0.97, 1.04)		0.713	
Comorbidities:								
DM	0.87 (0.65, 1.16)	0.342	0.88 (0.64, 1.22)	0.450	0.77 (0.47, 1.26)		0.296	
HTN	1.23 (0.85, 1.77)	0.264	1.74 (1.19, 2.53)	**0.004**	1.16 (0.66, 2.06)		0.602	
Atrial fibrillation	1.42 (0.65, 3.09)	0.381	1.92 (0.92, 3.97)	**0.080**	3.50 (1.52, 8.06)		**0.003**	
Obstructive sleep apnea	0.87 (0.50, 1.51)	0.629	0.65 (0.32, 1.30)	0.220	0.93 (0.36, 2.40)		0.876	
Long term anticoagulants	1.05 (0.42, 2.63)	0.917	1.65 (0.66, 4.15)	0.287	2.45 (0.80, 7.48)		0.116	
mFI-5	1.28 (0.58, 2.85)	0.539	2.37 (1.00, 5.60)	**0.050**	1.39 (0.39, 5.01)		0.615	
Current smoking	1.36 (0.77, 2.39)	0.291	1.14 (0.63, 2.06)	0.666	0.49 (0.15, 1.62)		0.243	
Hx of radiation	1.23 (0.32, 4.79)	0.766	1.40 (0.36, 5.46)	0.630	-		-	
Preop visual disturbances	1.61 (1.20, 2.16)	**0.001**	2.82 (2.00, 3.99)	**< 0.001**	5.27 (2.85, 9.77)		**< 0.001**	
Preop headache	1.27 (0.91, 1.78)	0.164	1.65 (1.16, 2.35)	**0.005**	2.39 (1.46, 3.91)		**< 0.001**	
Apoplexy	0.82 (0.44, 1.54)	0.544	0.95 (0.46, 1.97)	0.899	1.64 (0.67, 4.03)		0.278	
**Adenoma characteristics**								
Functional adenoma	0.52 (0.37, 0.72)	**< 0.001**	0.73 (0.48, 1.11)	0.139	0.54 (0.27, 1.08)		0.083	
ACTH	1.30 (0.74, 2.26)	0.361	1.38 (0.79, 2.42)	0.259	1.03 (0.43, 2.47)		0.954	
GH	0.24 (0.14, 0.42)	**< 0.001**	0.56 (0.27, 1.17)	0.125	0.34 (0.08, 1.40)		0.134	
PRL	0.43 (0.26, 0.71)	**< 0.001**	0.39 (0.19, 0.84)	**0.016**	0.28 (0.07, 1.15)		0.077	
Chiasm compression	1.65 (1.22, 2.24)	**0.001**	1.86 (1.28, 2.70)	**0.001**	4.57 (2.16, 9.67)		**< 0.001**	
Macroadenoma	1.95 (1.22, 3.10)	**0.005**	1.16 (0.66, 2.03)	0.615	1.99 (0.71, 5.60)		0.193	
Cavernous sinus invasion	1.65 (1.22, 2.23)	**0.001**	1.60 (1.15, 2.21)	**0.005**	2.07 (1.27, 3.36)		**0.003**	
Preop tumor volume (cm^3^)	1.38 (1.11, 1.72)	**0.004**	1.69 (1.39, 2.05)	**< 0.001**	1.69 (1.37, 2.08)		**< 0.001**	
**Operative characteristics**								
Surgery length (mins)	1.00 (1.00, 1.01)	**0.044**	1.01 (1.00, 1.01)	**< 0.001**	1.01 (1.01, 1.01)		**< 0.001**	
Intraop CSF leak	1.14 (0.85, 1.53)	0.373	1.48 (1.06, 2.06)	**0.022**	1.98 (1.18, 3.33)		**0.010**	
Use of lumbar drain	-	-	24.12 (7.08, 82.13)	**< 0.001**	8.82 (3.72, 20.91)		**< 0.001**	
Residual tumor volume (cm^3^)	1.10 (0.92, 1.32)	0.314	1.17 (1.01, 1.36)	**0.043**	1.21 (1.03, 1.41)		**0.022**	
EOR (%)	0.99 (0.98, 1.01)	0.283	0.98 (0.97, 1.00)	**0.018**	0.98 (0.96, 0.99)		**0.002**	
**Treatment outcomes**								
Postop complications:								
Postop CSF leak	-	-	34.28 (4.36, 269.58)	**< 0.001**	6.03 (1.72, 21.11)		**0.005**	
DI	0.98 (0.72, 1.32)	0.888	1.10 (0.79, 1.54)	0.570	1.29 (0.79, 2.11)		0.309	
Hyponatremia	1.44 (1.01, 2.04)	**0.043**	2.18 (1.53, 3.11)	**< 0.001**	3.26 (1.99, 5.32)		**< 0.001**	
Discharge disposition:								
Home	Ref		Ref		Ref			
Non home	5.77 (1.75, 19.03)	**0.004**	6.94 (3.38, 14.26)	**< 0.001**	7.31 (3.50, 15.29)		**< 0.001**	

Bold entries signify statistical significance, *p* < 0.05

Preop, preoperative; KPS, karnofsky performance score; BMI, body mass index; DM, diabetes mellitus; HTN, hypertension; mFI-5, modified frailty index-5; hx, history; intraop, intraoperative; EOR, extent of resection; postop, postoperative; DI, diabetes insipidus; DVT, deep vein thrombosis; PE, pulmonary embolism; UTI, urinary tract infection

Atrial fibrillation (*p* = 0.080 and *p* = 0.003) and preoperative headache (*p* = 0.005 and *p* < 0.001) were significant predictors of PLOS only when using the > 75th percentile and > 90th percentile definitions. Using these definitions, operative factors such as intraoperative CSF leak, use of a lumbar drain, residual tumor volume, and reduced EOR, along with postoperative CSF leak, were also associated with PLOS (*p* < 0.05). Factors such as non-White race (*p* < 0.001) and macroadenoma (*p* = 0.005) were predictors of PLOS when defined as > median, while hypertension (*p* = 0.004) and mFI-5 (*p* = 0.050) was significantly associated with PLOS defined as > 75th percentile. Conversely, Hispanic ethnicity and functional adenoma, including growth hormone and prolactin-secreting adenomas, were negatively associated with PLOS when defined as > median (*p* < 0.05).

### Multivariate analysis

**Fig. 1:**
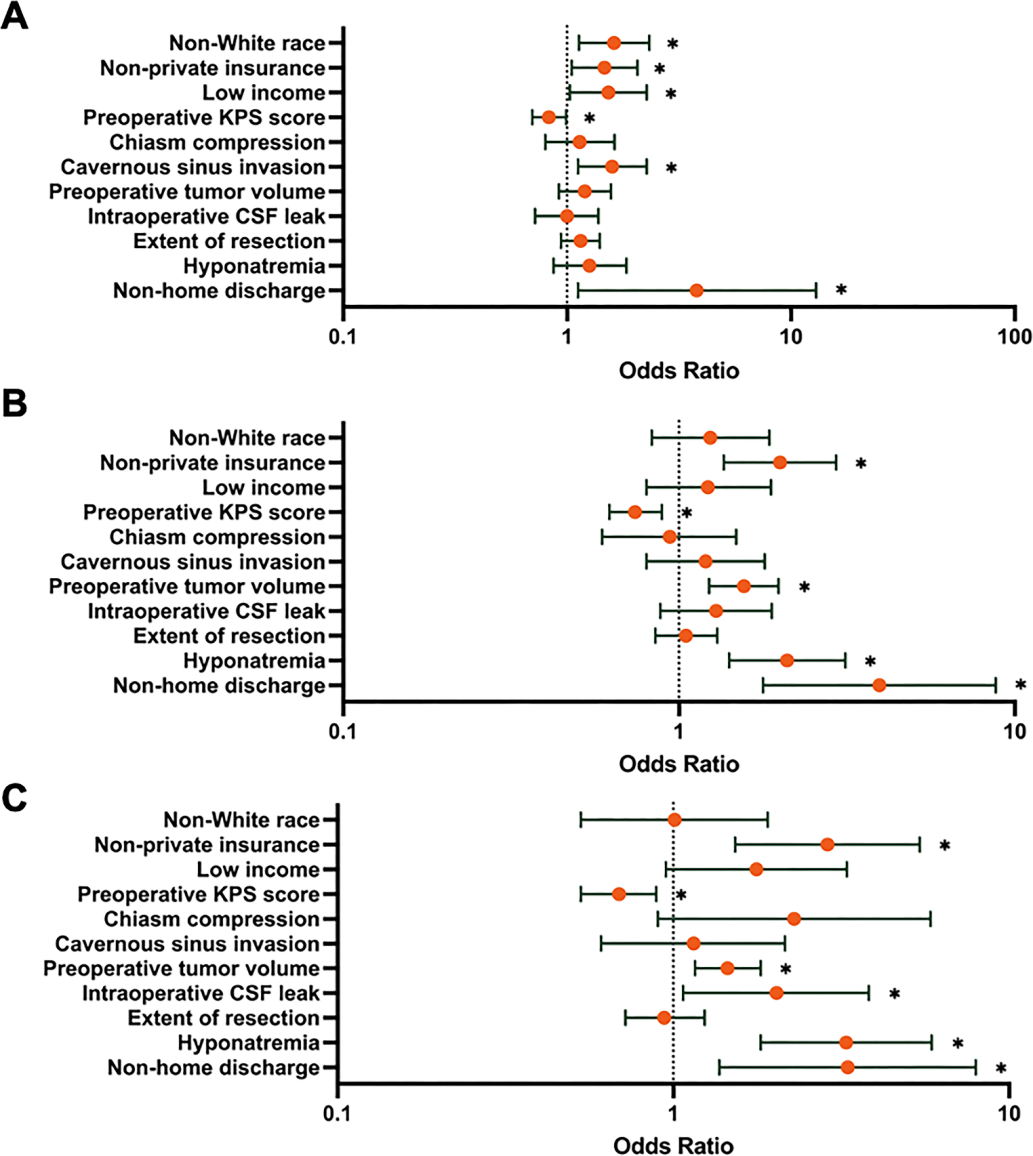
Forest plots showing results from multivariate logistic regression analyzing predictors of PLOS when defined as **A**) > median LOS, **B**) > 75th percentile of LOS, and **C**) > 90th percentile of LOS respectively. *p < 0.05

Multivariate logistic regression revealed non-private insurance (*p* = 0.026, *p* < 0.001, and *p* = 0.001) and non-home discharge (*p* = 0.033, *p* < 0.001, and *p* = 0.008) disposition to be significant predictors of PLOS across > median, > 75th percentile, and > 90th percentile definitions respectively (Table 4)(Fig. 1). Similarly, an increased preoperative KPS score (> median: *p* = 0.036, > 75th percentile: *p* = 0.001, and > 90th percentile: *p* = 0.004) was negatively associated with PLOS across all definitions. Increased preoperative tumor volume (*p* < 0.001 and *p* = 0.001) and postoperative hyponatremia (*p* < 0.001 and *p* < 0.001) predicted PLOS when defined as > 75th percentile and > 90th percentile, respectively. Finally, non-White race (*p* = 0.009) and low income (*p* = 0.035) were significant predictors of PLOS when defined as > median and intraoperative CSF leak (*p* = 0.029) was significant predictor when defined as > 90th percentile.

**Table 4:** Multivariate regression analysis of factors affecting PLOS as defined by varying definitions

PLOS definition	LOS > median		LOS > 75th percentile		LOS > 90th percentile	
Variables	Odds Ratio	*P* value	Odds Ratio	*P* value	Odds Ratio	*P* value
Race:						
White	Ref		Ref		Ref	
Non-white	1.62 (1.13, 2.33)	**0.009**	1.24 (0.83, 1.86)	0.293	1.01 (0.53, 1.91)	0.983
Insurance:						
Private	Ref		Ref		Ref	
Non-private	1.47 (1.05, 2.06)	**0.026**	2.00 (1.36, 2.94)	**< 0.001**	2.88 (1.53, 5.42)	**0.001**
Low income	1.53 (1.03, 2.27)	**0.035**	1.22 (0.80, 1.88)	0.353	1.77 (0.95, 3.29)	0.072
Preop KPS	0.83 (0.70, 0.99)	**0.036**	0.74 (0.62, 0.89)	**0.001**	0.69 (0.53, 0.89)	**0.004**
Chiasm compression	1.14 (0.80, 1.63)	0.469	0.94 (0.59, 1.48)	0.774	2.29 (0.90, 5.83)	0.083
Cavernous sinus invasion	1.59 (1.12, 2.27)	**0.010**	1.20 (0.80, 1.80)	0.380	1.15 (0.61, 2.15)	0.664
Preop tumor volume (cm^3^)	1.20 (0.92, 1.57)	0.172	1.56 (1.23, 1.98)	**< 0.001**	1.45 (1.16, 1.82)	**0.001**
Intraop CSF leak	1.00 (0.72, 1.38)	0.990	1.29 (0.88, 1.89)	0.195	2.03 (1.07, 3.82)	**0.029**
EOR (%)	1.15 (0.94, 1.40)	0.183	1.05 (0.85, 1.30)	0.633	0.94 (0.72, 1.24)	0.683
Hyponatremia	1.26 (0.87, 1.84)	0.226	2.10 (1.41, 3.13)	**< 0.001**	3.27 (1.82, 5.88)	**< 0.001**
Discharge disposition:						
Home	Ref		Ref		Ref	
Non home	3.80 (1.12, 12.96)	**0.033**	3.95 (1.78, 8.78)	**< 0.001**	3.31 (1.37, 7.96)	**0.008**

Bold entries signify statistical significance, *p* < 0.05

Preop, preoperative; KPS, karnofsky performance score; intraop, intraoperative; EOR, extent of resection

## Discussion

Policy initiatives such as bundled payments and pay-for-performance incentivize hospital leadership to focus on reducing the excessive utilization of hospital resources [23, 24]. These efforts also include reducing LOS [3]. Since intraoperative and immediate postoperative complications can prolong LOS, it is often considered a proxy for the quality of care provided to patients [25]. Consequently, efforts are being made to develop protocols that facilitate shorter hospital stays following surgery for pituitary adenomas [26, 27].

However, in non-neurosurgical literature, it has been found that up to 50% of the variation in extended LOS cannot be explained by hospital complication rates [3]. Moreover, the relationship between LOS and the effectiveness and efficiency of care is not always straightforward [2]. Since non-clinical factors, such as insurance status and discharge disposition, play a significant role in LOS, some authors do not support using LOS as a quality metric for efficient hospital management unless it is appropriately adjusted for non-clinical characteristics [2].

To complicate the issue further, there is no consensus among authors on what should be considered PLOS after ETPS [12, 14–17]. With this background, we report the impact of various clinical and non-clinical factors on LOS after ETPS using varying definitions for PLOS. To the best of our knowledge, our study is the only single-institution study highlighting the influence of non-clinical factors on LOS after ETPS, while controlling for clinical variables. We believe our study will help stimulate discussions within the neurosurgical community to work towards a consensus on the definition of PLOS and serve as a foundation for developing processes to reduce the influence of non-clinical factors on LOS after ETPS.

### Study overview

In our study cohort, the median LOS was 3 days, the 75th percentile of LOS was 4 days, and the 90th percentile was 6 days. We confirmed our primary hypothesis that predictors for PLOS are influenced by the definition used for PLOS.

Upon multivariate analysis, preoperative KPS was negatively associated with PLOS across all definitions. Non-clinical factors, such as non-private insurance and non-home discharge disposition, were also consistently associated with PLOS. Preoperative tumor volume and postoperative hyponatremia were significant predictors of PLOS using the > 75th percentile and > 90th percentile definitions but did not reach significance in the greater than median definition. Lastly, race and cavernous sinus invasion were significantly associated with PLOS > median, while intraoperative CSF leak was only significantly associated with PLOS > 90th percentile.

### Patient characteristics

Preoperative KPS was the only factor found to be statistically significant in both univariate and multivariate analyses across all three definitions used in our study. No other preoperative variable was found significant in multivariate analysis across all definitions. This suggests that a patient’s overall functional status and ability to perform activities of daily living directly impact their hospital stay duration. Regarding associated medical comorbidities, hypertension was significant only in univariate analysis when PLOS was defined as > 75th percentile. It was not statistically significant for other definitions or in multivariate analysis. Similarly, Vimawala et al. noted hypertension as a significant predictor in univariate analysis when PLOS was defined as the 90th percentile [16]; however, it was not significant in multivariate analysis. Contradicting our findings, one study did not find a significant impact of hypertension on PLOS defined as > 4 days [14]. BMI was not significantly associated with prolonged length of stay (LOS) under any definition used, which contrasts with findings from a previous study that identified a significant association between BMI and complications such as CSF leaks and infections—factors known to contribute to PLOS [16, 28].

Another comorbid condition found to be significant in univariate analysis was atrial fibrillation when PLOS was defined as > 75th and > 90th percentile. This finding is consistent with Vimawala et al. [16]. While other studies found obstructive sleep apnea on CPAP, bleeding disorders, and insulin-dependent diabetes mellitus to be significant predictors [15, 16], we did not observe any significant differences for diabetes mellitus, obstructive sleep apnea, or anticoagulant use in our cohort across any definitions. Our study lacks details on the type of diabetes, obstructive sleep apnea, and bleeding disorders, which could help explain these differences. We found mFI-5 to be significant only in univariate analysis when PLOS was > 75th percentile (4 days). Similar to our findings, Khalafallah et al. found that each point increase in mFI-5 was associated with an increase in LOS by 1.65 days [29]. Additionally, Vasan et al. found Charlson Comorbidly Index to be significant in univariate analysis using the definition of PLOS > median (3 days) [12]. However, we did not find mFI-5 to be significant in univariate analysis when using the definition of PLOS used by Vasan et al.

Preoperative visual disturbances were found to be significant in univariate analysis for all PLOS definitions. In contrast, preoperative headache was only significant at the 75th and 90th percentile definitions in univariate analysis. None of the other studies have evaluated the impact of these symptoms on PLOS.

### Adenoma characteristics

Cavernous sinus invasion, preoperative tumor volume, and chiasm compression were significantly associated with PLOS in univariate analysis across all PLOS definitions. However, in multivariate analysis, cavernous sinus invasion was the only tumor characteristic that significantly associated with PLOS defined as > median LOS. When the definition of PLOS was changed to > 75th and > 90th percentile, cavernous sinus invasion was no longer significant on multivariate analysis. Instead, preoperative tumor volume emerged as a significant predictor of PLOS under these definitions. Similar to our findings, Devarajan et al. found that maximum tumor dimension was a significant predictor of PLOS defined as > 4 days in multivariate analysis [14]. This author also noted that previous radiation therapy a was significant factor in univariate analysis [14]. However, we were unable to confirm this finding in our study. This discrepancy may be due to the higher percentage of patients with prior radiation therapy in their study cohort compared to ours (2.9% vs. 1.23%) [14].

### Operative characteristics

Upon univariate analysis, only duration of surgery was found to be a significant factor across all definitions of PLOS. No other intraoperative factor was significant when using a definition of PLOS > median. However, upon changing the definition to > 75th and > 90th percentile, intraoperative CSF leak, EOR, residual tumor volume, and use of lumbar drain were found to be significantly associated with PLOS. Other authors also noted duration of surgery and intraoperative CSF leak to be significantly associated with PLOS on univariate analysis [14, 16]. On the other hand, on multivariate analysis, none of the intraoperative factors were found to be significant across all definitions. One factor, intraoperative CSF leak, was a significant predictor of PLOS when defined as > 90th percentile. Our findings align with those of Vimawala et al., who also found that intraoperative CSF leak was the only intraoperative factor significantly associated with PLOS > 90th percentile [16].

### Postoperative outcomes

Hyponatremia was identified as a significant clinical factor associated with PLOS in univariate analysis across all definitions used. However, upon multivariate analysis, it was a significant predictor of PLOS only for the definitions of > 75th and > 90th percentiles. Other single-institution studies did not analyze this parameter but noted postoperative CSF leak as a significant factor in both univariate and multivariate analyses for PLOS > 4 days and > 90th percentile. In our study, postoperative CSF leak was not a significant factor with PLOS > median but was significant on univariate analysis for PLOS > 75th and > 90th percentiles. Upon multivariate analysis, postoperative CSF leak was significant only when using the PLOS > 90th percentile definition. Contrary to other studies, we did not find diabetes insipidus to be a significant predictor of PLOS [14, 16]. Studies in neurosurgical literature also show non-neurosurgical complications such as pulmonary embolism, deep vein thrombosis, and urinary tract infections to impact LOS [30, 31]. We found the rates of only deep vein thrombosis to be significantly different between non-PLOS and PLOS when defined as > 75th percentile. However, we did not find a significant difference in these variables within our cohort in any of other definitions evaluated.

### Non-clinical predictors of PLOS

Along with preoperative KPS, non-private insurance and non-home discharge disposition were the only factors that were consistently significant across all definitions on both univariate and multivariate analyses. Similar to our results, in a study based on the NIS database, Vasan et al. found Medicaid to be a significant factor associated with PLOS defined as > median stay (3 days) [12]. A study from non-neurosurgical literature, involving 313,144 patients from the National Trauma Database, noted that patients with Medicaid had a significantly longer LOS compared to those with private insurance [2]. This study also noted that discharge disposition had the greatest effect on LOS, with patients discharged to nursing homes and rehabilitation facilities experiencing longer LOS [2]. This data clearly highlight role of non-clinical parameters in patients’ hospital stays.

Additionally, low income was significant across all definitions on univariate analyses but only for PLOS > median for multivariate analyses. This correlates with another study that found lower income to be associated with frailty [30]. Presumably, patients with lower income face greater barriers to assessing timely healthcare, causing to delayed hospital visits, increased frailty, and more advanced disease presentation which in turn contributes to PLOS. When using the definition of PLOS > median, we found that race was significantly associated with PLOS in both univariate and multivariate analyses, while ethnicity was significant only in univariate analysis. Both race and ethnicity were not statistically significant in either univariate or multivariate analyses when the definitions of PLOS > 75th percentile and > 90th percentile were used. Using the NIS database and defining PLOS as > median (3 days), Vasanth et al. showed that female, Black, and Hispanic patients were more likely to experience PLOS [12]. Similarly, an analysis of the NSQIP database involving 11,510 patients undergoing craniotomies for tumors found that African American and Hispanic patients experienced PLOS [31].

### Limitations and strengths

Our study is inherently limited by its retrospective nature. To overcome these limitations, we included only patients with complete records available. Additionally, it is a single-institution study. Therefore, validation in larger cohorts and multi-institutional collaborations is needed.

Despite these limitations, to the best of our knowledge, we report the largest single-center series showing the predictors of PLOS in patients undergoing ETPS for PA and how these predictors vary by the definition of PLOS used. Compared to generic database efforts to characterize predictors of PLOS, our study has significant granular data regarding preoperative characteristics, tumor characteristics, and postoperative outcomes which are not always readily available in various databases. As such, we believe that our results highlight clinical predictors common across various definitions of PLOS as well as the significant role of non-clinical variables in PLOS. None of the single-institution studies have evaluated the impact of these non-clinical factors on PLOS.

## Conclusion

Upon multivariate analysis, we found that non-clinical factors such as insurance status and discharge disposition, as well as the patient’s overall functional status as determined by the preoperative KPS score were significantly associated with PLOS across all definitions used. Changing the definition of PLOS altered the impact of patient presentation, adenoma characteristics, operative characteristics, and postoperative outcomes in both univariate and multivariate analyses. This study demonstrates that factors related to the primary disease and its treatment do not significantly influence PLOS consistently across varying definitions of PLOS. Instead, insurance status, preoperative functional status, and discharge disposition play a crucial role in PLOS. These findings align with conclusions from non-neurosurgical literature, suggesting that PLOS should be appropriately adjusted for patient’s functional status and non-clinical factors before it is used as a quality metric for evaluating hospitals and physicians performing ETPS.

## Data Availability

No datasets were generated or analysed during the current study.
